# Stable Cycling of All-Solid-State Lithium Batteries Enabled by Cyano-Molecular Diamond Improved Polymer Electrolytes

**DOI:** 10.1007/s40820-024-01415-3

**Published:** 2024-06-17

**Authors:** Yang Dai, Mengbing Zhuang, Yi-Xiao Deng, Yuan Liao, Jian Gu, Tinglu Song, Hao Yan, Jin-Cheng Zheng

**Affiliations:** 1 Department of Chemical Engineering, Shanghai University, Shangda Road 99, Shanghai, 200444 People’s Republic of China; 2 Department of Physics, Xiamen University, Xiamen, 361005 People’s Republic of China; 3 Experimental Center of Advanced Materials School of Materials Science and Engineering, Beijing Institute of Technology, Beijing, 100081 People’s Republic of China; 4 Department of Physics and Department of New Energy Science and Engineering, Xiamen University Malaysia, 43900 Sepang, Malaysia

**Keywords:** 1-Adamantanecarbonitrile (ADCN), Poly (ethylene oxide), All-solid-state batteries, Interfacial stability, High voltage

## Abstract

The interfacial instability of the poly(ethylene oxide) (PEO)-based electrolytes impedes the long-term cycling and further application of all-solid-state lithium metal batteries. In this work, we have shown an effective additive 1-adamantanecarbonitrile, which contributes to the excellent performance of the poly(ethylene oxide)-based electrolytes. Owing to the strong interaction of the 1-Adamantanecarbonitrile to the polymer matrix and anions, the coordination of the Li^+^-EO is weakened, and the binding effect of anions is strengthened, thereby improving the Li^+^ conductivity and the electrochemical stability. The diamond building block on the surface of the lithium anode can suppress the growth of lithium dendrites. Importantly, the 1-Adamantanecarbonitrile also regulates the formation of LiF in the solid electrolyte interface and cathode electrolyte interface, which contributes to the interfacial stability (especially at high voltages) and protects the electrodes, enabling all-solid-state batteries to cycle at high voltages for long periods of time. Therefore, the Li/Li symmetric cell undergoes long-term lithium plating/stripping for more than 2000 h. 1-Adamantanecarbonitrile-poly(ethylene oxide)-based LFP/Li and 4.3 V Ni_0.8_Mn_0.1_Co_0.1_O_2_/Li all-solid-state batteries achieved stable cycles for 1000 times, with capacity retention rates reaching 85% and 80%, respectively.

**Supplementary Information:**

The online version contains supplementary material available at 10.1007/s40820-024-01415-3.

## Highlights

- Additive of 1-adamantanecarbonitrile is used to strengthen the poly(ethylene oxide) based solid polymer electrolytes (SPEs).
- LiF-based integral cathode/SPE and Li/SPE interfaces are generated.
- The NMC811/Li all-solid-state performs stable cycle at 45 °C (1000, 80%).

**Supplementary Information:**

The online version contains supplementary material available at 10.1007/s40820-024-01415-3.

## Graphical abstract

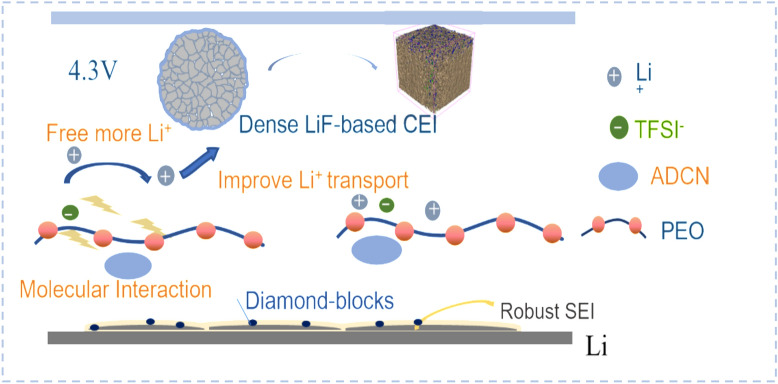

## Introduction

All-solid-state lithium polymer batteries combined with solid electrolytes to replace the liquid electrolytes and separators of traditional lithium-ion batteries [1–3] are regarded as the potential candidates for the next-generation energy storage applications due to their attractive advantages [4–14]. These virtues include but not limited to (1) highly thermal stability, composed of an intrinsically safe solid polymer electrolyte that is non-leakable, toxic and flammable, (2) excellent flexibility and processability, (3) relative suitability for lithium metal anodes with mechanical compatibility and improving the energy density, and (4) separated by SPEs, crosstalk behaviors can be effectively suppressed, prolonging the cycling-life. Solid polymer electrolyte (SPE), as the core part of the battery, largely determines the performance of the battery. PEO-based SPEs have industrial dominance and excellent processing applicability, and have been widely commercialized in the fields of electric vehicle batteries and large-scale energy storage [4, 10]. One of the main shortcomings is the limited conductivity of Li^+^ (10^−8^–10^−6^ S cm^−1^ at room temperature), have been partially overcome and improved to 10^−4^ S cm^−1^ at near-room temperature [11, 15], tamed by a second phase adding (liquid, ceramic, and polymer composite), salt engineering or additives [4–8, 16, 17].

Although the PEO-based electrolyte is relatively suitable for lithium metal anodes, lithium dendrite growth and side reactions will still occur at the electrolyte/lithium metal anode interface after long-term cycling, leading to interface degradation and performance deterioration [10, 18, 19]. To suppress this degeneration, various SPE-enhancing strategies have been applied, such as cross-linking [20, 21], fluorination of polymer matrix, and addition of oxides, inert or conductive ceramics, salts, MOFs, nanodiamonds [22–25] into the electrolyte membranes. Impressively, by introducing high-modulus and inactive nanodiamonds into the electrolyte membranes, the lifetime of lithium symmetric batteries is effectively increased, and the dendrite growth is significantly suppressed [25]. Nevertheless, these effective strategies may only act on the anode without contributing to the stability of the cathode interface. Furthermore, the addition of the second phase may lead to the heterogeneity of the electrolyte membranes [16, 17, 22].

Another serious challenge is the unstable nature of the PEO-based electrolytes at high voltage (> 4 V vs. Li/Li^+^) [10, 15]. Polymer electrolytes are thought to degenerate under high voltage, producing parasitic reactions. This serious drawback makes them difficult to couple to the high voltage cathodes (> 4 V), such as nickel-rich, lithium-rich and spinel. In addition, it is reported that PEO-based SPEs have difficulty passivating the nickel-rich layered cathodes LiNi_*x*_Mn_*y*_Co_*z*_O_2_ (NCM, *x* > 0.5, *x* + *y* + *z* = 1), which are regarded as the promising cathodes for high-energy batteries applied in EVs. Therefore, considerable efforts have been made to improve the intrinsic and interfacial stability of the SPEs, such as cathode materials-coating [26], salt-engineering [17, 27], dual-layered design and polymer composition adjustment of the SPEs [28, 29], enabling the all-solid-state batteries (ASSBs) coupled with the NCM cathodes to cycle to 4.2–4.4 V. Unfortunately, the lifetime of NCM/Li ASSBs remains elusive due to severe interfacial degradation [7, 20, 27, 30–38]. Moreover, most of the approaches focus on one-sided interface, hardly meeting the demand of the long-term cycling for the ASSBs, which require both interfaces of the anode/SPE and cathode/SPE are compatible.

1-adamantanecarbonitrile (ADCN), as an important intermediate for valuable drugs, contains a stable “building blocks”—C_10_H_15_- used to synthesize diamond [39, 40] and the high voltage-stabilized functional group -CN. It is worth noting that succinonitrile (CN(CH_2_)_2_CN) is widely used to plasticize PEO-based electrolyte [41, 42]. In addition, ADCN is also added to the polymer as a stabilizer to protect the polymer from ultraviolet radiation [43, 44].

Inspired by the research on nanodiamond additive [19], we adopted ADCN as an additive to enhance SPE and improve the cycling performance of the ASSBs. 1-Adamantanecarbonitrile dissolves well in SPE, whereas adamantane is difficult to dissolve. The ADCN can also act as plasticizer to improve ion transport in SPEs. The ‘molecular diamond block’ C_10_H_15_- enhances the stability of the SPEs, thereby triggering the growth of a stable SEI and inhibiting the lithium dendrite growth of lithium dendrites. In addition, the CN- group-based ADCN will stabilize the cathode/SPE interface, thereby achieving stable cycling of NCM/Li ASSBs. Therefore, the ADCN-enhanced PEO-based electrolyte exhibits an improved ionic conductivity of 1.44 × 10^−4^ mS cm^−1^ at 45 °C, which is 7 times higher than the baseline SPE. As a result, long-term lithium deposition/stripping over 2000 h can be achieved for Li/Li batteries assembled using ADCN-based SPE. In addition, ASSB was used in conjunction with LiFePO_4_ and ADCN-incorporated PEO-based electrolyte to achieve stable cycling at 0.3 C at 45 °C for 1000 times with a capacity retention of 85%. Impressively, the high voltage 4.3 V NCM/ ADCN-SPE /Li ASSB exhibits excellent cycling capability (1000, 80%). Therefore, our work demonstrates an effective approach to enhance interfacial stability on both anodic and cathodic sides mediated by SPE, thereby enabling long-term stable cycling of high voltage ASSBs using PEO-based electrolytes.

## Experimental Section

### Preparation of the PEO-Based SPEs

Preparation of the PEO-Based SPEs: The membrane were prepared by a classic solvent-casting approach. 1 g PEO (Mw ≈ 600,000) and 0.466 g LiTFSI were dissolved in ~ 22 mL anhydrous acetonitrile (AN, Aladdin) and strongly stirred at room temperature overnight to generate a homogeneous solution. ADCN (Aladdin) with different mass of 20, 50, and 100 was put into the above solution, respectively. The procedures are operated in a glove-box (Ar filled, O_2_·H_2_O < 0.1 ppm, Mikrouna). The resulted solutions were poured onto a polytetrafluoroethylene (PTFE) plate and dried under vacuum at 80 °C for 36 h. The obtained membranes are recorded as LiTFSI/P(EO)_14_/ADCN-1(1 wt% PEO), LiTFSI/P(EO)_14_/ADCN-2(5 wt% PEO), and LiTFSI/P(EO)_14_/ADCN-3(10 wt% PEO).

### Structure and Material Characterization

The SEM images were recorded by scanning electron microscope (SEM) (Hitachi S-4800). X-ray diffraction (XRD, Bruker D8 Advance) was used to determine the structure of SPE membranes and cathodes. The Fourier-transform infrared spectroscopy (FTIR) were recorded on Thermo Scientific Nicolet iS20 (400–2000 cm^−1^). X-ray photoelectron spectroscopy (XPS) were recorded on a Thermo Scientific K-Alpha with an Al Kα radiation (1486.5 eV). The elemental depth distribution information was obtained by Time of Flight-Secondary Ion Mass Spectrometer (TOF–SIMS, PHI nano TOF II, ULVAC-PHI Inc. Japan). Stress–strain experiments were performed by a universal testing machine (UTM5305) at a rate of 10 mm min^−1^. TOF–SIMS measurements including sample preparation and testing process were carried out according to the standard T/SHPTA 035–2023. For the TOF–SIMS measurement, the interface of cycled NCM811 electrodes were detected. Bi^3+^ beam (30 kV) was used as the primary beam to detect the samples and the sputter etching was performed using an Ar^+^ beam (3 kV 100 nA) to obtain the desired depth profile. The area of analysis was 50 × 50 μm^2^ while the sputtering area was 400 × 400 μm^2^. All the XPS and TOF–SIMS tests were performed in a home-made air-free capsule between the glovebox and instruments.

The Li^+^ conductivity of the SPEs was tested by using SS (stainless steel) |SPEs|SS cell from 20 to 80 °C. Electrochemical impedance spectroscopy (EIS) measurements were recorded on a Solartron 1260 + 1287 (10 mV, 1 MHz to 0.01 Hz). The linear sweep voltammetry (LSV) was scanned at 1 mV s^−1^, within the potential range from 2.5 to 6 V.

### Preparation LFP and NMC811 Cathodes

LFP or NMC811, conductive carbon, SPE-based composition, and PVDF (75/10/10/5 in weight) were carefully blended and stirred in *N*-methyl-2-pyrrolidone (NMP) to form a homogeneous slurry. The slurry was then casted on an aluminum foil (C-coated) and then vacuum-dried at 80 °C for 24 h to form the resulting electrodes, with active mass loading of 2–3 mg cm^−2^. The ASSB was assembled with cathode, SPE, and Li metal in 2032 cell in above-mentioned glove-box. For cycled material characterization, the cycled cathode and lithium metal were carefully dissembled and rinsed with dimethyl carbonate (DMC) and dried in glove-box.

### Calculate Method

The density functional theory (DFT) computation have been performed using the CP2K program [45]. All the geometry optimizations were calculated by the semiempirical method MNDO (Modified Neglect Diatomic Overlap). The dissociation energy was calculated as follows: *ΔE* = *E*_2_ − *E*_1_, where *E*_1_ is the LiTFSI or LiTFSI-ADCN molecular system before dissociation, *E*_2_ is the energy possessed by molecular fragments obtained after dissociation reactions based on different possible optimized structure configurations.

## Results and Discussion

### Characterization of SPEs

**Fig. 1:**
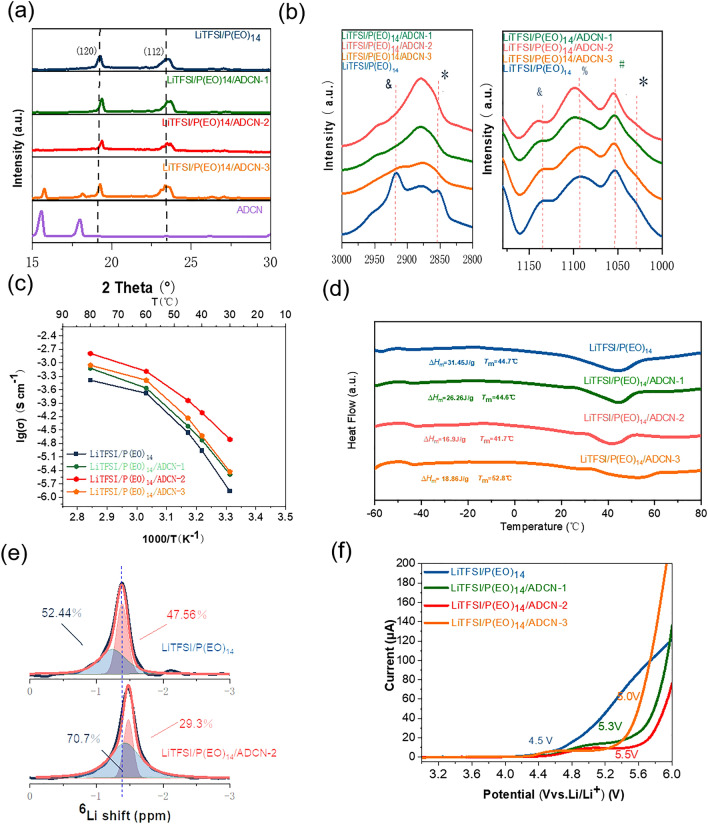
**a** XRD patents, **b** FTIR spectra (3000–2800 cm^−1^and 1180–1000 cm^−1^), **c** Arrhenius plots, **d** DSC profiles of SPE membrane, **e** High-resolution solid-state ^6^Li MAS NMR spectra and **f** LSV plots of the of the various SPE membranes (1 mV s^−1^)

The prepared PEO-based and ADCN-PEO-based membranes showed a lucent and homogenous appearance in the pictures. Furthermore, SEM images of smooth and dense surfaces and EDS images with uniform elemental distribution (Fig. S1), reveal that both LiTFSI and ADCN can be well ‘immersed’ into the polymer matrix. Figure 1a plots the XRD patterns of the SPEs. Two representative peaks at 19.1° and 23.6° in pure LiTFSI/P(EO)_14_ electrolyte can be indexed as (120) and (112) crystalline phases of PEO-based electrolyte [8, 16, 17]. The shape and height changes of the peak upon addition of ADCN indicate the interaction and mediation of the ADCN-LITFSI-PEO. However, when the addition amount increases to *x* = 10 wt% (PEO-ADCN_*x*_), the ADCN phase appears, demonstrating that the solvation of ADCN is highly incomplete. The FTIR spectra were further used to study molecular interactions, as shown in Fig. 1b. The peaks located at 1000–1160 cm^−1^ can be ascribed to the symmetrical stretching model (*ν*_*s*_) of the C–O–C, which exhibits triplet peaks at 1146, 1086, and 1060 cm^−1^. These peaks can be attributed to the stretching of the coordinated C–O–C [7, 27, 46]. Upon addition of ADCN, the position and shape changes of the peak were red-shifted, indicating strong interaction of the ADCN and EO fragments. This is due to the strong interaction between the existing electron-withdrawing species –CN and the lone pair of electrons of –EO–, which weakens the coordination. The bands around 2900 cm^−1^ can be assigned to the C–H stretching, which is so sensitive to the extent of LiTFSI complexation [47, 48]. It can be observed that the baseline SPE exhibit doublet peaks, suggesting the existence of salt-based crystalline-like structure. After adding the ADCN, the doublet peaks disappear and immerges into one peak. This phenomenon can be attributed to the strong interaction of the ADCN with the LiTFSI, fostering the association of the salt. Furthermore, the peaks at 720–760 cm^−1^ (Fig. S1e) can be divided into free TFSI^−^ anion components and aggregated ion pairs [7, 27, 46]. It is obvious that the aggregated ion-pairs component is enhanced upon addition of ADCN, which highlights the significant interaction of ADCN-TFSI^−^. Among the four samples, LiTFSI/P(EO)_14_/ADCN-2 showed the highest content of aggregated ion pairs.

The Arrhenius plot, shown in Fig. 1c, reveals the improvement of the ionic conductivity of SPEs by using different amounts of ADCN. The ionic conductivity of SPE is significantly enhanced due to the obvious interaction of ADCN-LITFSI-PEO. The conductivity of the SPE (LiTFSI/P(EO)_14_/ADCN-1) with the addition of only 1 wt% is significantly enhanced compared to that of baseline. In SPEs, the ionic conductivity with the addition of 5 wt% (LiTFSI/P(EO)_14_/ADCN-2) ADCN presented the highest 0.144 × 10^−3^ mS cm^−1^ at 45 °C (Fig. 1c), which was 7 times higher than the baseline SPE at the same temperature. However, a high content of 10 wt% leads to a decrease in ionic conductivity, which may be due to the inhibition of ion transport by incompletely incorporated particles. Owing to the interaction of ADCN (Fig. 1d), the *T*_*g*_, and *T*_*m*_ for the Bi-based SPEs reduce. Among them, LiTFSI/P(EO)_14_/ADCN-2 possesses the lowest *χ*_*c*_ (ratio of crystalline) (Table S1), revealing the high content of amorphous part. The ADCN may act as the plasticizer, decreasing the crystalline and thus improving the Li^+^ ion-transport. Nevertheless, since the uncomplete dissolution of the ADCN with high content, the effect would be weakened [11]. The mechanical strength is another essential metric. As illustrated in Fig. S1f, the tensile strength of the baseline is 1.3 MPa. With the addition of ADCN, the strengths have been enhanced. This enhancement may be attributed to the molecular interactions of the ADCN with –EO– units, which crosslinks the polymer matrix. Among them, the LiTFSI/P(EO)_14_/AdCN-2 has the highest mechanical strength, reaching 5.1 MPa. The typical membranes of LiTFSI/P(EO)_14_ and LiTFSI/P(EO)_14_/ADCN-2 are also explored by the ^6^Li NMR spectra (Fig. 1e). The peaks can be fitted into the two parts, which are the Li^+^ coordinated by PEO/TFSI^−^ (red peaks) and the more free Li^+^ (blue peaks), respectively. The results present the higher content of the mobile-Li^+^ part (blue peak, 70.70%) for the LiTFSI/P(EO)_14_/ADCN-2, while the LiTFSI/P(EO)_14_ only contains that of 52.44% [30]. An obvious peak-up-field-shifting for the LiTFSI/P(EO)_14_/ADCN-2 compared to the LiTFSI/P(EO)_14_ demonstrates the decreasing of the interaction between Li^+^ and polymer matrix, thus freeing more Li^+^ ions in the SPE. These results agree with that of the FTIR. Due to the strong "binding" effect of ADCN-TFSI^−^ and TFSI^−^, free Li^+^ can be released well. Therefore, the typical Li^+^ transference number (*t*_Li_^+^) of LiTFSI/P(EO)_14_/ADCN-2 is as high as 0.38 compared with the baseline of 0.25, indicating lower anion migration capability in ADCN-based electrolytes (Fig. S2). Furthermore, since ADCN stabilizes the anion, the linear sweep voltammetry (LSV) curve (Fig. 1f) shows a significant improvement in anode stability from 4.5 V for baseline to 5.0–5.5 V for the ADCN-based electrolytes [29, 35]. Therefore, these excellent advantages of the ADCN-based electrolytes demonstrate their potential application in lithium metal ASSBs. It will be interesting to evaluate the performance of symmetric cells (such as Li/SPE/Li) and asymmetric cells (e.g., ASSB) containing ADCN-based SPEs, which will be presented in the following sections.

### Li Metal/Li Metal Symmetric Cell Performance

**Fig. 2:**
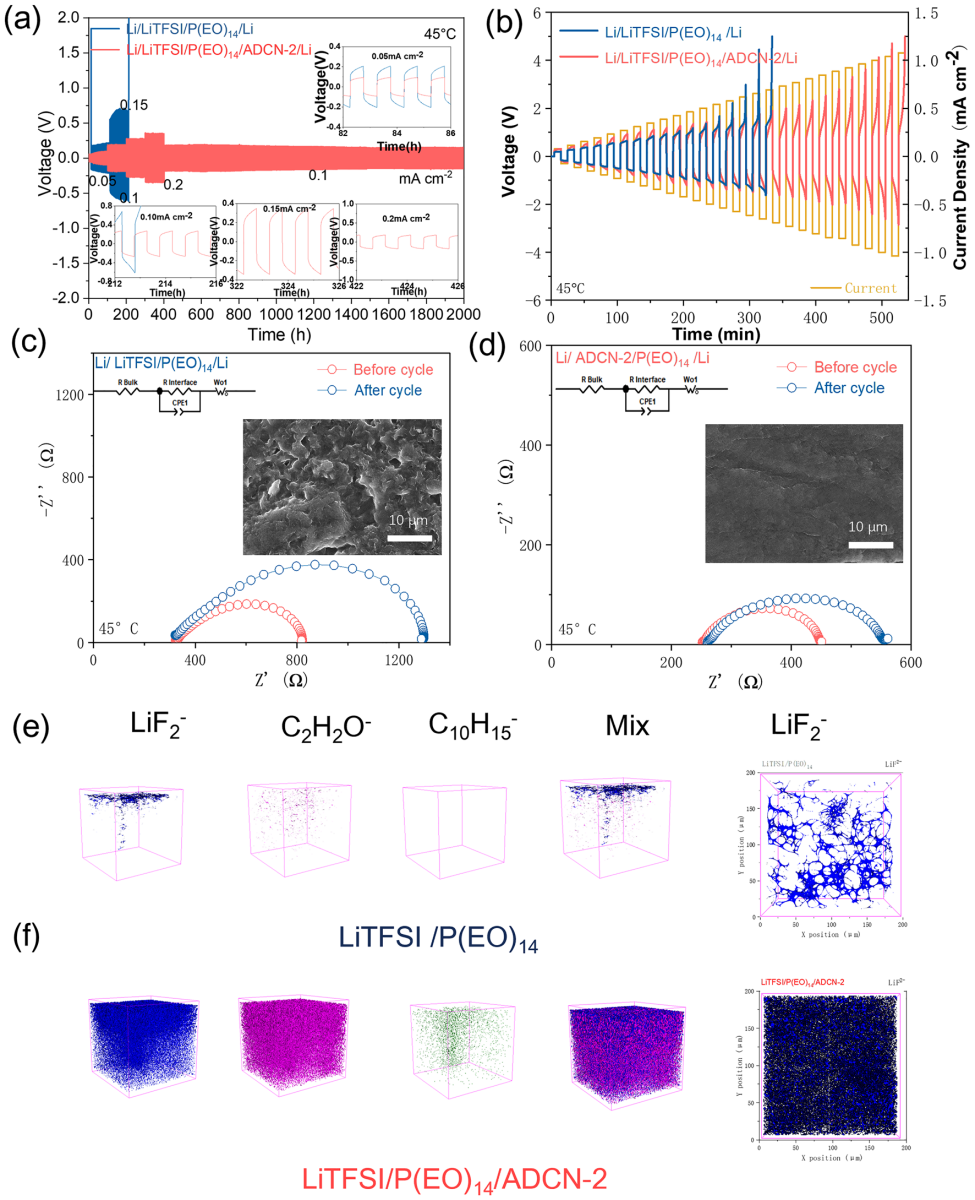
**a** Long-term cycling profiles, and **b** Critical current density test curves of the LiTFSI/P(EO)_14_ and LiTFSI/P(EO)_14_/ADCN-2 based Li//SPE//Li symmetric cells, respectively. EIS spectra of the Li//SPE//Li cells before and after 200 cycles for the **c** LiTFSI/P(EO)_14_ and **d** LiTFSI/P(EO)_14_/ADCN-2-based Li//SPE//Li symmetric cells, respectively. The inset pictures are the SEM images of the corresponding lithium anode after cycling. **e** 3D-view of the typical species of LiF_2_^−^, C_2_H_2_O^−^, C_10_H_15_^−^and 2D view of the LiF^−^ for the cycled lithium metal with LiTFSI/P(EO)_14_ and **f** LiTFSI/P(EO)14/ADCN-2

To further investigate the anodic compatibility of SPEs with lithium metal anodes, the membranes were sandwiched in symmetric Li/SPE/Li cells and evaluated at 45 °C. In Fig. 2a, the cell using LiTFSI/P(EO)_14_/ADCN-2 maintained stable long-term lithium plating/delithiation for more than 2000 h and could even withstand a high current density of 0.2 mA cm^−2^. This excellent performance may originate from the dendrite suppression ability and compatible interface of ADCN-based electrolytes. In sharp contrast, the cell using LiTFSI/P(EO)_14_ only operated for a short period of 213 h before failing due to a short circuit [9]. As illustrated in Fig. 2b, the critical current density of LiTFSI/P(EO)_14_/ADCN-2 SPE is 1.1 mA cm^−2^, while that of the baseline is 0.7 mA cm^−2^. This can be attributed to the higher strength of the membrane and interfacial stability. In fact, the SEM image (Fig. 2d, inset) shows that after long-term cycling, the surface of the decomposed lithium anode based on ADCN is smooth and dense without any cracks and dendritic structures, while the cyclic lithium anode based on LiTFSI/P(EO)_14_ showed cavities and mossy structures, indicating poor interfacial compatibility (Fig. 2c, inset). The Li/SPE/Li cells are also investigated by EIS (Fig. 2c, 2d). The curves can be fitted by the *R*_bulk_ (intercept of *x*-axis) and *R*_interface_ (semicircle at high and medium frequencies), and the equivalent circuits are indicated in inset of Fig. 2c, 2d. It has been reported that the *R*_interface_ plays an essential role in the performance of the Li/Li batteries [49, 50]. Indeed, the cell with baseline exhibiting a large charge-transfer resistance (*R*_interface_) increase from 800 to 1300 Ω, while the cell with ADCN-enhanced SPE showed a much smaller increment from 450 to 560 Ω.

Powerful TOF–SIMS further explores the lithium anode/SPE interface. Studying by TOF–SIMS, the depth-profiles and 3D-reconsturted typical species of LiF_2_^−^, and C_2_H_2_O^−^, C_10_H_15_^−^ are illustrated in Fig. S3 and Fig. 2e, respectively. It can be detected that the SEI film of the cycled anode with LiTFSI/P(EO)_14_/ADCN-2 are rich in LiF_2_^−^, which is formed from the LiF with dense structure. Impressively, the rigid ‘diamond blocks’ (C_10_H_15_^−^) are uniformly distributed across the SEI film, which can effectively inhibit the growth of lithium dendrites. Therefore, the formation of a dense electrically insulating SEI is conducive to Li^+^ diffusion and can well protect the negative electrode from degradation [25, 51]. In sharp contrast, the baseline SEI presents sparse appearance and lacks a dense LiF layer, making it difficult to prevent anode degradation.

### All-Solid-State Li-Metal Batteries

**Fig. 3:**
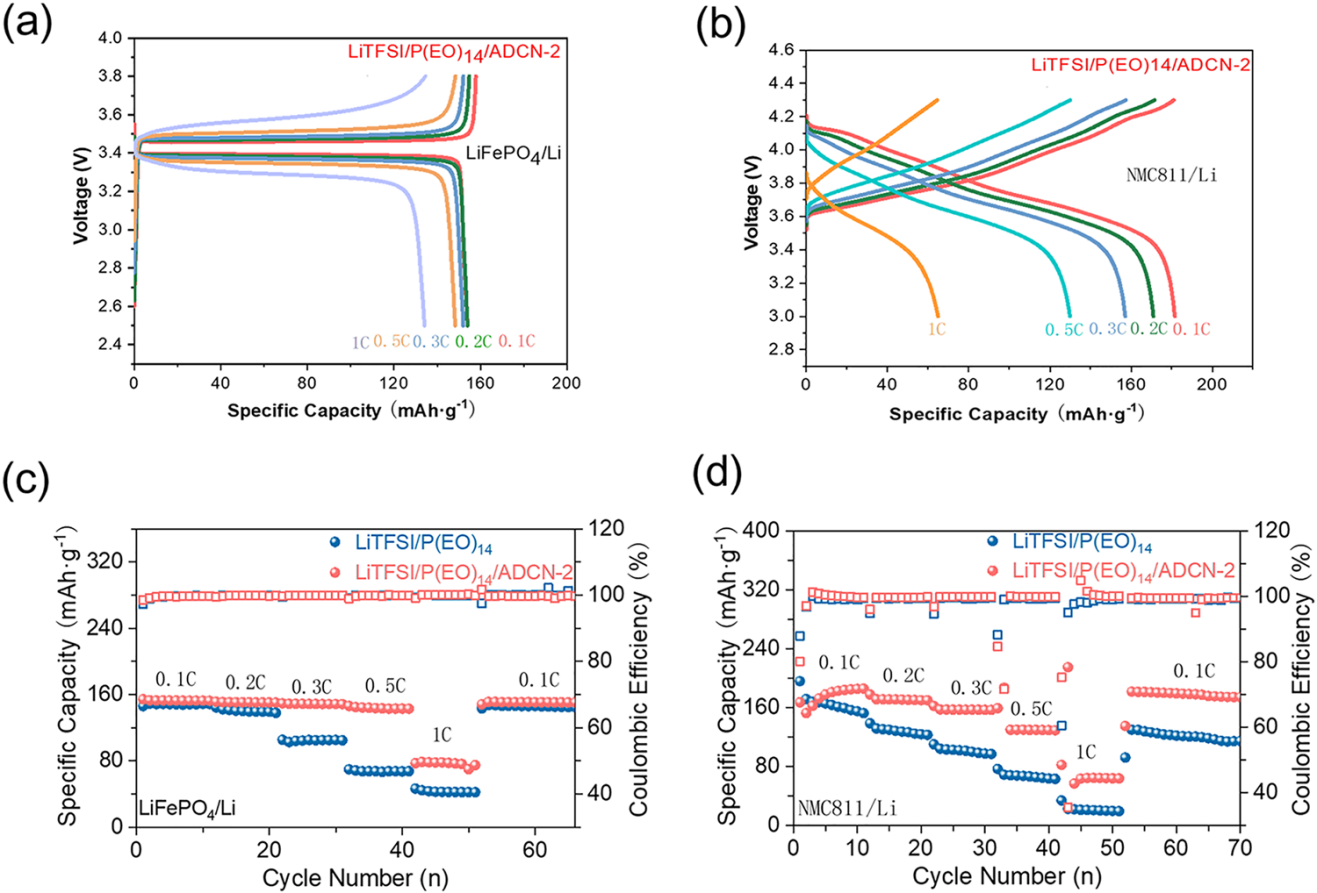
Charge–discharge profiles of Charge–discharge curves of the **a** LFP/LiTFSI/(PEO)_14_/ADCN-2/Li and **b** NMC811/LiTFSI/(PEO)_14_/ADCN-2/Li at various rates. Rate performance of **c** LFP/LiTFSI/(PEO)_14_/ADCN-2/Li and **d** NMC811 /LiTFSI/(PEO)_14_/ADCN-2/Li under cycling

**Fig. 4:**
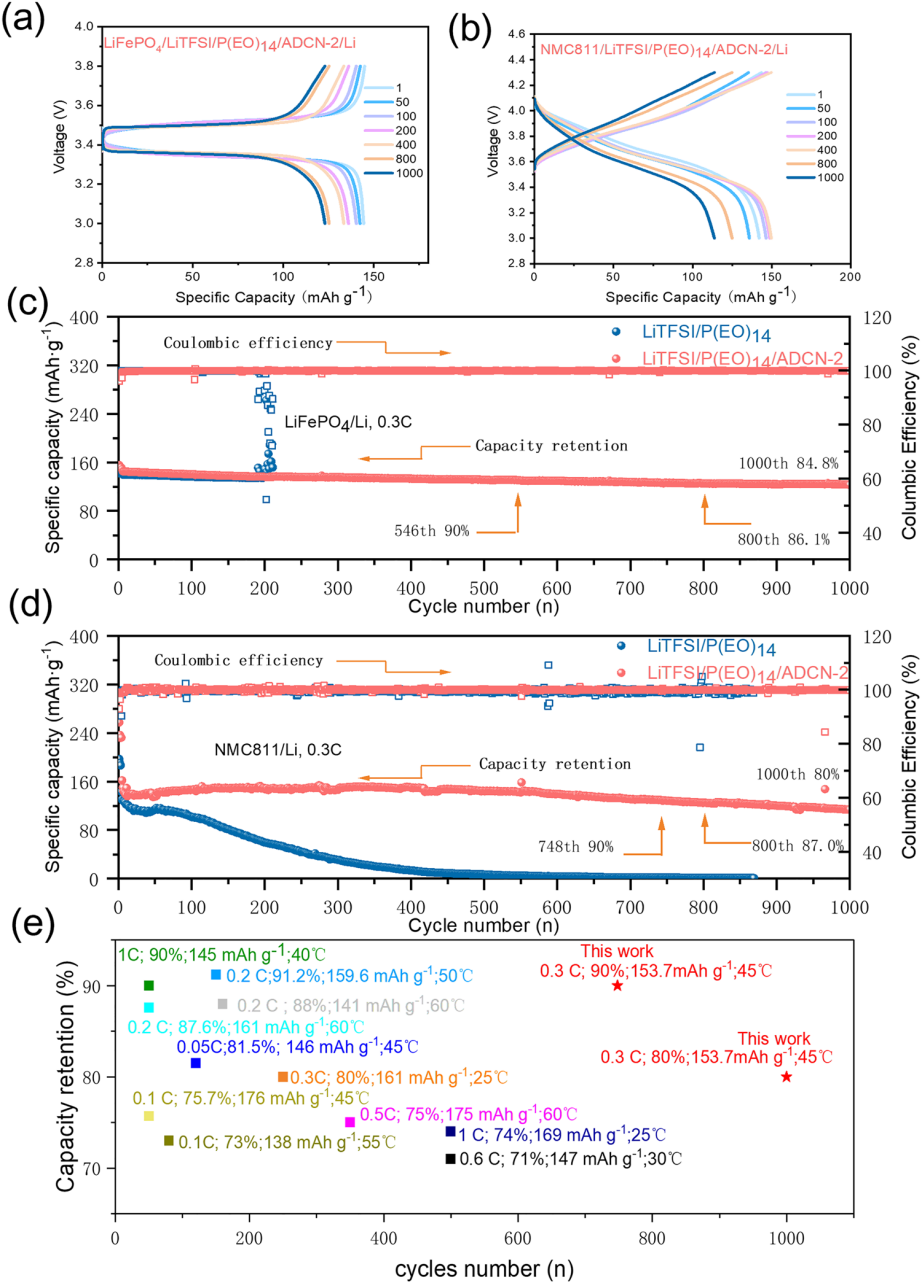
Charge–discharge profiles of Charge–discharge curves of the **a** LFP/LiTFSI/(PEO)_14_/ADCN-2/Li and **b** NMC811 /LiTFSI/(PEO)_14_/ADCN-2/Li at various cycles. Cycling performance of **c** LFP/LiTFSI/(PEO)_14_/ADCN-2/Li and **d** NMC811 /LiTFSI/(PEO)_14_/ADCN-2/Li. **e** Comparison of the recent reported PEO-based NCM/Li ASSBs with our results

To verify the practice ability of ADCN-based SPEs, ASSBs based on two representative cathodes, LiFePO_4_ (LFP) and LiNi_0.8_Co_0.1_Mn_0.1_O_2_ (NMC811), were paired and evaluated at 45 °C, respectively. Figures 3a, S4a, and 3c compare the performance of LiTFSI/P(EO)_14_ and LiTFSI/P(EO)_14_/ADCN-2 in LFP/Li cells, respectively. LiTFSI/P(EO)_14_/ADCN-2-based cells obtained discharge capacities of 152.85,150.78,148.82,144.76, and 78.64 mAh g^−1^ at 0.1, 0.2, 0.3, 0.5, and 1 C, respectively, indicating a good capacity recovery when return to 0.1 C. In comparison, LiTFSI/P(EO)_14_ based cell exhibits inferior performance in terms of rate, specifically delivering only 47 mAh g^−1^ at 1 C. Furthermore, the ‘gaps’ between the charge and discharge curves can be clearly observed (Fig. S5). The curves at 1 C show that much smaller polarity for the LiTFSI/P(EO)_14_/ADCN-2-based cell 296.5 mV and higher capacity than that of the baseline cell (LiTFSI/P(EO)_14_) 479 mV, revealing the superior kinetic behavior of ADCN-based electrolytes. Importantly, the LiTFSI/P(EO)_14_/ADCN-2-based cell exhibits unique cycling capability, with a capacity retention of 84.8% after 1000 cycles at 0.3 C, which surpasses that of 60% for the baseline after 200 cycles. Figures 3b, S4b, and 3d display the rate performance of the NCM811/SPE/Li cells at 45 °C. The LiTFSI/P(EO)_14_/ADCN-2-based cell delivers charge capacities of 181, 171, 157.4, 130, and 64.5 mAh g^−1^, at rate of 0.1, 0.2, 0.3, 0.5, and 1 C, respectively. In stark contrast, the baseline cell suffers from severe capacity attenuation above 0.2 C, only exerting a capacity of at 161.11, 126.56, 96.9, 67.7, and 25.52 mAh g^−1^, at rate of 0.1, 0.2, 0.3, 0.5, and 1 C, respectively. Furthermore, the LiTFSI/P(EO)_14_/ADCN-2-based cell exhibits well-defined charge–discharge profiles, while the baseline exhibits the distorted and highly polarized curves above 0.3 C. Figures S6a and 5a, c compare the cycling performance of the LFP/Li-based cells with LiTFSI/P(EO)_14_ and LiTFSI/P(EO)_14_/ADCN-2, respectively. Impressively, the LFP/Li cell with LiTFSI/P(EO)_14_/ADCN-2 shows stable long-term cycling with 84.8% capacity retention at the 1000th cycle, while the LFP/Li cell with the baseline can only last 200 cycles and eventually end with a sudden loss of capacity. Long-term cycling performance is an important metric for NCM811/Li ASSBs (Figs. S6b and S5b, d), especially with high cut-off potential. In fact, as shown in Fig. 4d, the 4.3 V cell based on LiTFSI/P(EO)_14_/ADCN-2 can achieve more than 1000 long-term cycles, with an excellent capacity retention of 80%, highly overtaking that of 55% for the baseline after 200 cycles. The outstanding rate and cycling capability of the LiTFSI/P(EO)_14_/ADCN-2-based cells could be mainly attributed to the robust interfaces generated by the cathode/SPE and lithium anode/SPE. Figure 4e and Table S2 compare the electrochemical performance of the NCM/Li ASSB reported in the literature, including other solid-state electrolyte systems (e.g., halides, hydroborates, and thiophosphate-based electrolytes), and our results are competitive for voltage cut-off, long-term cycling, and capacity retention [7, 20, 27, 29–38]. Furthermore, the *R*_*ct*_ in the baseline-based EIS of cell changes significantly after 200 times. While cell based on LiTFSI/P(EO)_14_/ADCN-2 exhibits almost overlapping curves, indicating that the formed interfaces are stable during cycling.

**Fig. 5:**
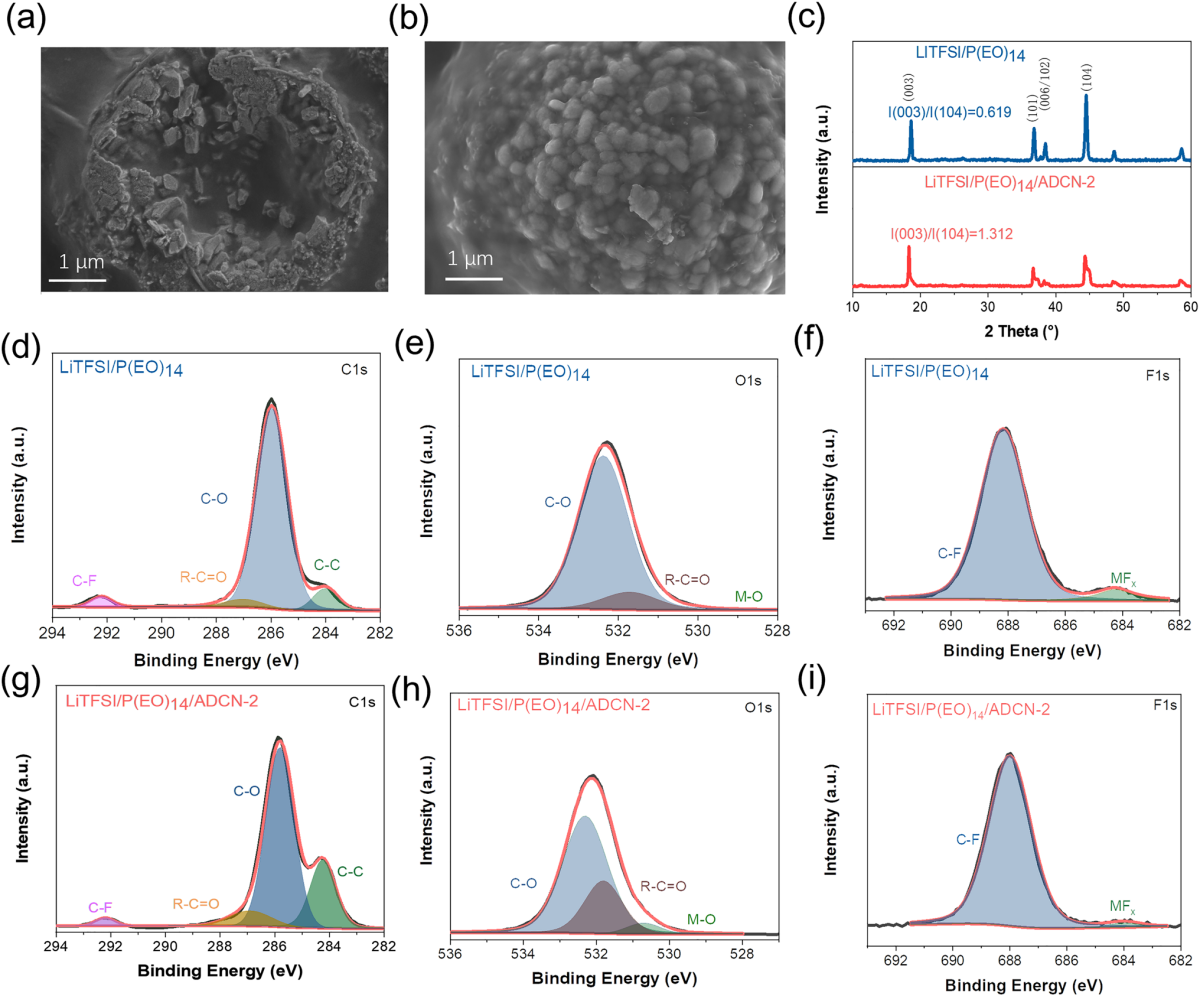
SEM images for Particles of the cycled cathode materials with **a** LiTFSI/P(EO)_14_ and **b** LiTFSI/P(EO)_14_/ADCN-2, **c** XRD profiles of the cycled cathodes, XPS spectra of **d** C 1*s*, **e** O 1*s*, and **f** F 1*s* for the cycled cathodes with LiTFSI/P(EO)_14_ and **g** C 1*s*, **h** O 1*s*, and **i** F 1*s*

Since the cathode/SPE interface plays a vital role in the battery-performance, we further employed the XRD, SEM, XPS, and TOF–SIMS to study the interfacial properties of NCM/SPE [5, 7, 16]. Figure 5a, b presents the SEM images of the cycled cathodes with baseline and LiTFSI/P(EO)_14_/ADCN-2, respectively. The cycled cathode with baseline exhibits cracked and incomplete secondary particles with a thick surface layer, which may be caused by strains and stresses generated by the incompatible interface. For comparison, a cycled cathode with the LiTFSI/P(EO)_14_/ADCN-2 maintains clear and intact secondary particles, demonstrating the well-shielded cathode and durable interface formed. The XRD curves of the cycled cathodes are shown in Fig. 5c. The ratio of structure factor with different index may provide useful information about the crystal structure details and even orbital population [52, 53]. As an indicator of the degree of degradation of the cathode material, the lower I_(003)_/I_(104)_ ratio reveals a higher content of rock salt structure in the cycled cathode [54], which is the by-product of the cathode material during the cycling process. The I_(003)_/I_(104)_ ratio for the LiTFSI/P(EO)_14_/ADCN-2 paired cathode is 1.312, exceeding that of the baseline-based cathode (0.619), highlighting the interfacial stability of the cathode paired with the LiTFSI/P(EO)_14_/ADCN-2. The XPS spectra (C 1*s*, O 1*s*, and F 1*s*) are recorded in Fig. 5d–i. In C 1*s*, the C–O species are formed from the degradation of the PEO matrix. MF_*x*_ species are produced by the deterioration of the cathode [54]. Therefore, the content of the C–O and MF_*x*_ species in the cycled cathode is higher compared to the baseline, indicating the presence of incompatibility and interfacial instability during cycling. In sharp contrast, lower C–O and MF_*x*_ compositions can be observed in the LiTFSI/P(EO)_14_/ADCN-2-based cathode, demonstrating higher compatibility and stability of the interface. In addition, the content of the M–O species (transition metals derived from cathode materials) in O 1*s* indicates the stability of the interface.

The M–O (Transition Metal Oxides) component is barely visible in the baseline-based cathode, indicating that the CEI film is too thick to detect the M–O signal [55] (lattice O^2−^). That can be attributed to the highly degraded and incompatible interface of the cathode, resulting in thick CEI growth. For comparison, M–O species can be clearly detected, showing the formation of a thin but strong interface. Furthermore, representative ion fragments distribution of LiF_2_^−^, C_2_H_2_O^−^, and C_10_H_15_^−^ were recorded by TOF–SIMS to study the CEI composition. According to the depth profile and 3D-views, the main composition of the CEI of the baseline-based cathode is highly sparse and heterogeneous, while the main component of the LiTFSI/P(EO)_14_/ANCN-2-based cathode is highly dense with LiF_2_^−^, which may be derived from the LiF and the organic F containing species [17, 27]. And the LiF is electronically insulating, preventing the cathode from degrading under high voltage. Also, C_10_H_15_^−^ species are uniformly distributed, which helps to construct the robust and insulated interface. Furthermore, the combination of ‘rigid’ (inorganic) and ‘soft’ (organic) species in the CEI may release the strain and stress during charge/discharge, which contributes to the long-term cycling of high-voltage ASSBs of NCM/Li.

In addition, ADCN, especially the –C≡N groups in ADCN also contribute to the cathodic stability. According to calculated results of HOMO–LUMO energy for various components in the SPEs (Fig. S8a), the HOMO energy of ADCN (− 7.96 eV) is 1.18 eV lower than that of PEO (− 6.78 eV), indicating that ADCN has excellent oxidation resistance. Thus, the presence of ADCN in a high voltage environment can reduce the rate of parasitic reactions and by-products [56]. FTIR spectra also show a lower peak intensity of about 1080 cm^−1^ (–C–O–C– vibration) in the cycled NMC811 cathode with LiTFSI/P(EO)_14_/ADCN-2 compared to that with the LiTFSI/P(EO)_14_. This result indicates the content of the –EO– segments on the surface of cathode, which suppresses the parasitic reactions by employing ADCN. Furthermore, the XPS N 1*s* spectra present higher content of Li_*x*_NO_*y*_ in the cycled cathode with LiTFSI/P(EO)_14_/ADCN-2 compared to that with LiTFSI/P(EO)_14_. Similar to LiF, Li_*x*_NO_*y*_ has been reported to protect the cathode and provide pathways for the Li^+^-diffusion through the CEI, ensuring fair ion transport [56].

**Fig. 6:**
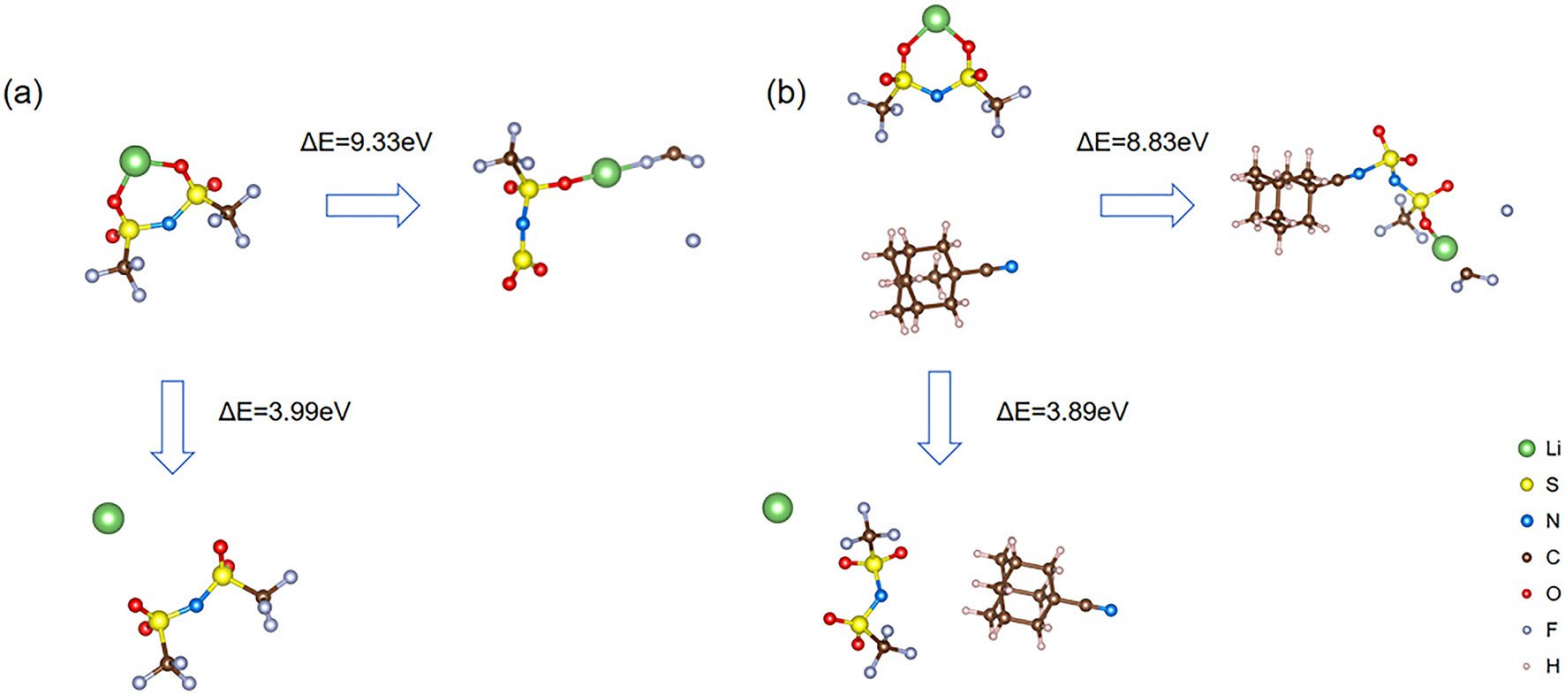
Dissociated energy of LiTFSI before and after adding ADCN

**Fig. 7:**
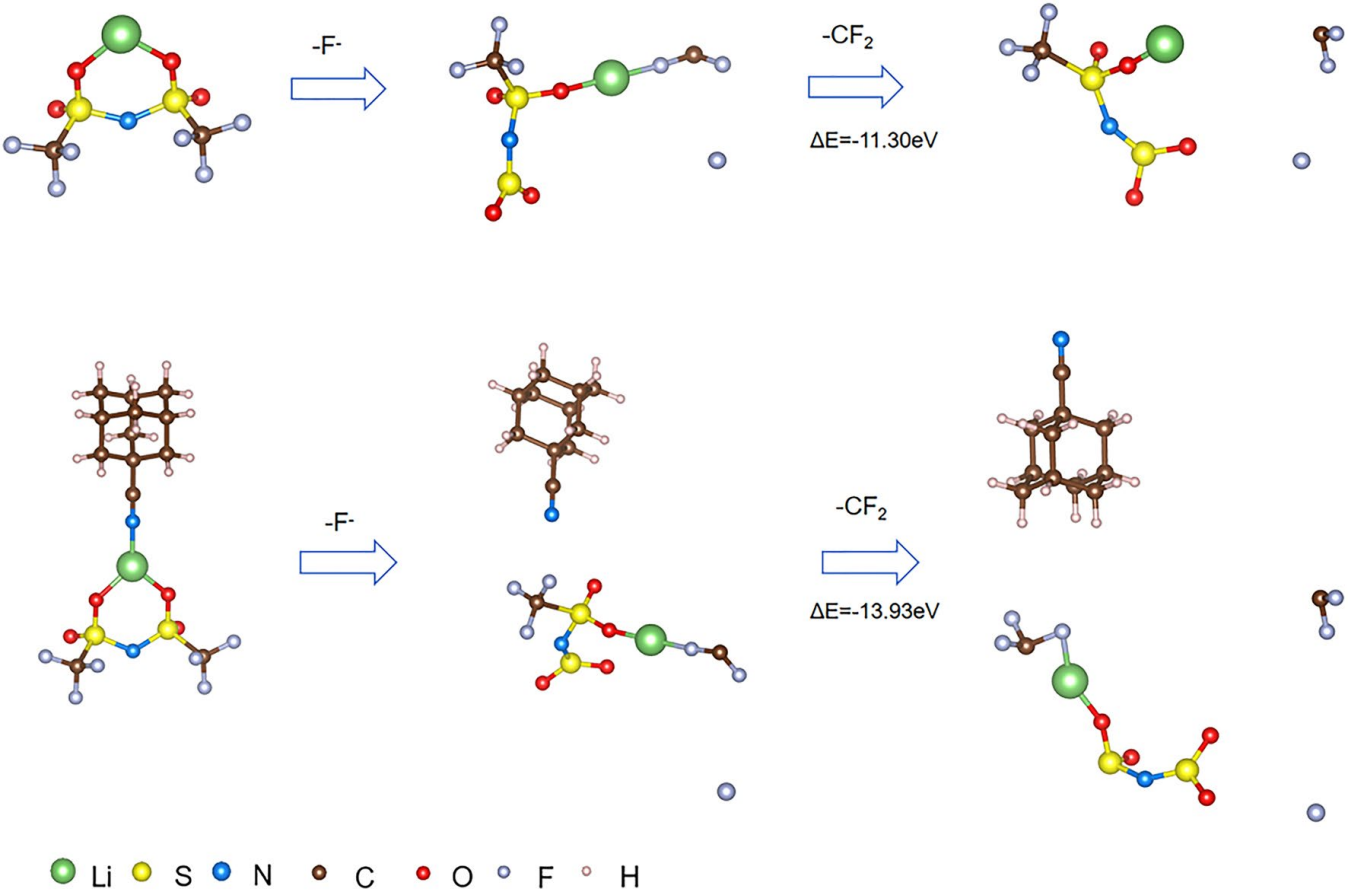
Proposed decomposing processes of the LiTFSI and LiTFSI + ADCN systems

In our systems, LiF plays critical roles in the SEI and CEI films to shield the lithium anode and cathode (especially at high voltage), the possible formation mechanisms of LiF are further elucidated. Therefore, we employed the DFT computation using CP2K package to investigate the mechanisms in Figs. 6 and 7. LiF is mainly originated from the decomposition of the LiTFSI, especially, F is from the -CF_3_ of the LiTFSI.

The formation of LiF in our system comes from Li^+^ and F^−^, which are decomposed by the LiTFSI, and the F^−^ mainly originates from the -CF_3_ group. In order to explore the possible impact of the ADCN additive on the formation of LiF in lithium salts, SEI and CEI, the energy required for a LiTFSI molecule to gradually detach one Li^+^ ion, one F^−^ ion and then –CF_2_ group before and after adding ADCN was calculated.

The calculation results show that after adding ADCN, when ADCN and LiTFSI molecules are closed to each other but not yet connected, the energy required for a LiTFSI molecule to detach from a Li^+^ ion is reduced from 3.99 to 3.89 eV, and the energy required to detach an F^−^ ion is decreased from 9.33 to 8.83 eV. This shows that ADCN promotes the decomposition of Li^+^ and F^−^ ions in LiTFSI, which is beneficial to the formation of LiF in SEI and CEI (Fig. 6).

In particular, we also found that when the –CN end of ADCN is connected to LiTFSI, if an F^−^ ion first detaches from LiTFSI, the energy required to further detach a CF_2_^−^ from LiTFSI is reduced by 2.63 eV (Fig. 7). It is shown that the presence of ADCN contributes to the further decomposition of -CF_3_ in LiTFSI, so more F^−^ ions can combine with free Li^+^ ions to form LiF. And the generated LiF-rich CEI and SEI can significantly protect the lithium anode and cathode, respectively, enabling long-term longevity.

## Conclusion

In summary, we have demonstrated an effective additive, ADCN, which contributes to the excellent performance of the PEO-based electrolytes. Due to the strong mediation of the ADCN to the polymer matrix and anions, the coordination of the Li^+^-EO is weakened, and the bondage of anion is enhanced, which helps to improve Li^+^ conductivity and electrochemical stability. More importantly, the ADCN can promote the formation of LiF in the SEI and CEI, and the Li_*x*_NO_*y*_ in CEI, which is beneficial to the interfacial stability and enables long-term cycling of LFP/Li and high voltage NCM811/Li. Therefore, the Li/Li symmetric cell can with stand long-term lithium plating/stripping for more than 2000 h. The 1000-long-term-cycle of LFP/ LiTFSI/P(EO)_14_/ADCN-2/Li and 4.3 V NMC811/ LiTFSI/P(EO)_14_/ADCN-2/Li ASSBs can be achieved, with an impressive capacity retention of 85% and 80%, respectively. Compared to the former solutions of one-sided interface, hardly meets the demand of the long-term cycling for the ASSBs. Our work addressed on both interfaces of the anode/SPE and cathode/SPE, making them compatible.

Therefore, our work provides a potential avenue for designing high-performance ASSBs with long span life.

## Supplementary Information

Below is the link to the electronic supplementary material.

Supplementary file1 (PDF 1139 kb)
