# Supplementary material for: Stable Cycling of All-Solid-State Lithium Batteries Enabled by Cyano-Molecular Diamond Improved Polymer Electrolytes
Source: Nanomicro Lett. 2024 Jun 17;16:217. doi: 10.1007/s40820-024-01415-3 (PMC11182998; doi:10.1007/s40820-024-01415-3)
Supplement: Supplementary file 1 — Supplementary file1 (PDF 1139 kb) [file 40820_2024_1415_MOESM1_ESM.pdf]

Supporting Information for

Stable Cycling of All-Solid-State Lithium Batteries Enabled by Cyano-Molecular Diamond Improved Polymer Electrolytes

Yang Dai[1], Mengbing Zhuang[1], Yi-Xiao Deng[2], Yuan Liao[1], Jian Gu[1,*], Tinglu Song[3,*], Hao Yan[1,*], Jin-Cheng Zheng[2,4,*]

[1]Department of Chemical Engineering, Shanghai University, Shangda Road 99, Shanghai, 200444, P. R. China

[2]Department of Physics, Xiamen University, Xiamen, 361005, P. R. China

[3]Experimental Center of Advanced Materials School of Materials Science & Engineering, Beijing Institute of Technology, Beijing, 100081, P. R. China

[4]Department of Physics and Department of New Energy Science and Engineering, Xiamen University Malaysia, Sepang 43900, Malaysia

* Corresponding authors. E-mail: hao-yan@shu.edu.cn (Hao Yan); gujian@shu.edu.cn (Jian Gu); song@bit.edu.cn (Tinglu Song); jczheng@xmu.edu.cn (Jin-Cheng Zheng)

S1 Symmetric Li/SPEs/Li Cell Assembly

The 2032 typed symmetric Li/SPEs/Li (φ=10 mm) cells were performed on a Solartron 1260+1287 workstation employing EIS measurements at 45 °C (AC amplitude of 10 mV, 1 MHz to 0.01 Hz). The plating/stripping profiles were recorded by a Land CT2001A.

The Li+ transference number (t_{Li^+}) of LiTFSI/P(EO)$_{14}$ and LiTFSI/P(EO)$_{14}$/ADCN-2 membrane was measured in a symmetric Li cell at 45 °C with a DC polarization of 10 mV. The t_{Li^+} was calculated using the equation:

$$t_{Li^+} = \frac{I_S(\Delta V - I_0 R_0)}{I_0(\Delta V - I_0 R_S)} \quad \text{(S1)}$$

Here, I_0 and I_s are the initial and steady-state currents, ΔV is the applied potential. R0 and Rs indicate the charge-transfer resistance before and after the polarization of the cell, respectively.

The HOMO and LUMO energies were performed under the Gaussian and plane-wave (GPW) approach [S1] using B3LYP as exchange and correlation potential functional [S2], and double-zeta valence plus polarisation (DZVP) basis set in combination with Geodecker-Teter and Hutter(GTH) pseudopotentials were employed [S3].

S2 Supplementary Figures and Tables

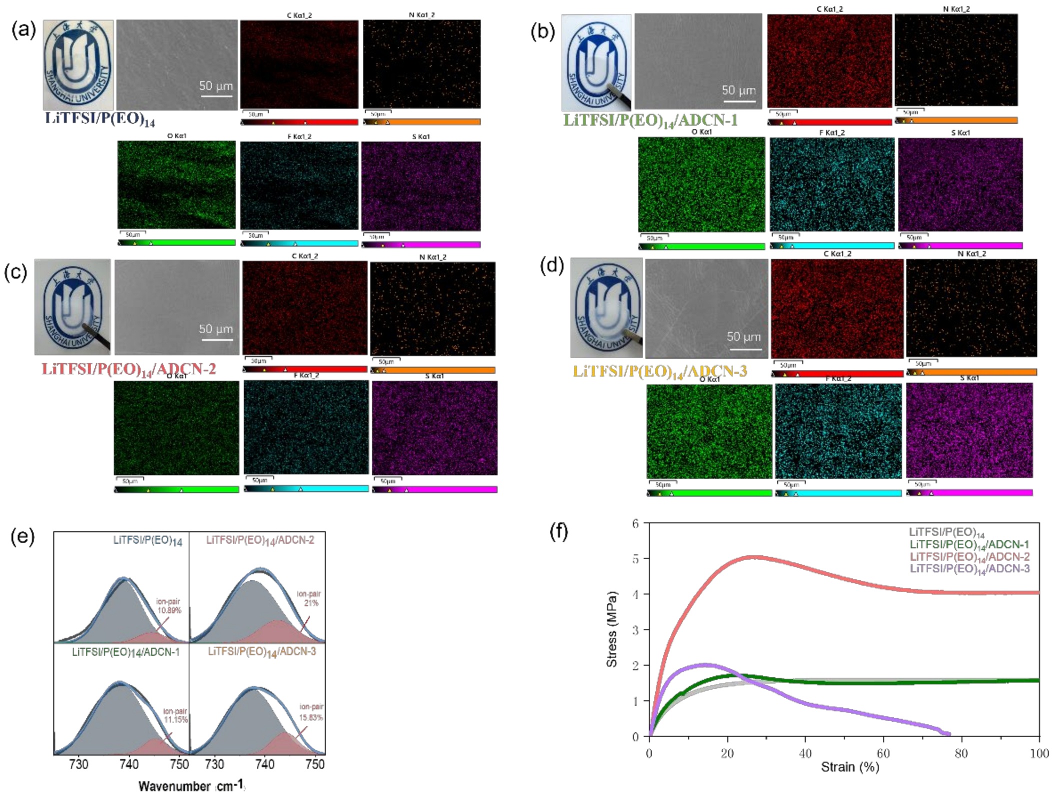

Fig. S1 Photo, SEM and EDS images of the SPEs (**a**) LiTFSI/P(EO)14 , (**b**) LiTFSI/P(EO)14/ADCN-1,(**c**) LiTFSI/P(EO)14/ADCN-2, and (**d**) LiTFSI/P(EO)14/ADCN-3. (**e**) FTIR spectra at the range of 728-752 cm^{-1} of the various SPEs. (**f**) The stress-strain profiles of various SPEs

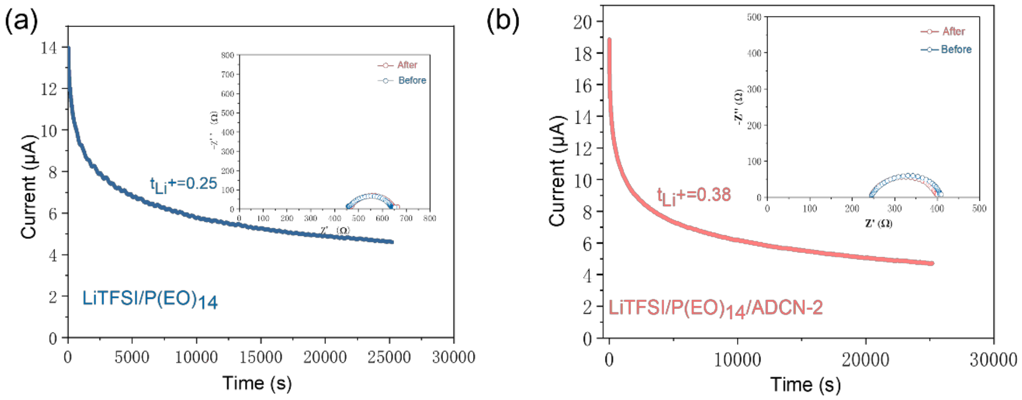

Fig. S2 Chronoamperometry profiles of Li//Li cells with (**a**) LiTFSI/P(EO)14 , and (**b**) LiTFSI/P(EO)14/ADCN-2, The insets are the corresponding EIS before and after polarization

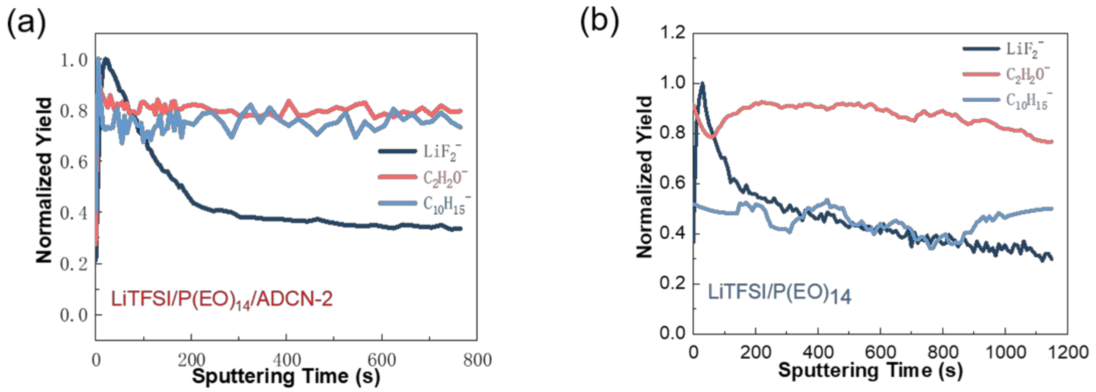

Fig. S3 TOF-SIMS depth profiles of the typical species and corresponding 3D-views for the cycled lithium anodes in the symmetric cells with LiTFSI/ $P(EO)_{14}$ (**a**) LiTFSI/$P(EO)_{14}$/ADCN-2 (**b**) after cycling, respectively

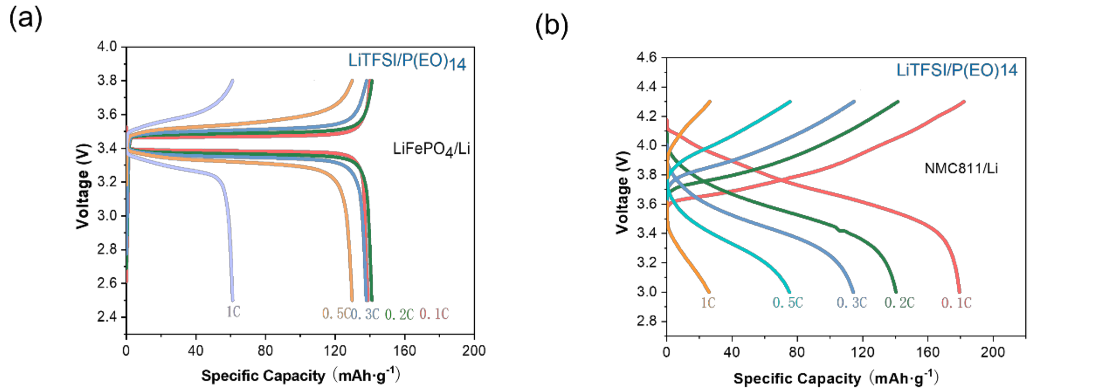

Fig. S4 Charge-discharge curves of the (**a**) LFP/LiTFSI/$(PEO)_{14}$/Li (**b**) NMC811 /LiTFSI/$(PEO)_{14}$/Li at various rates

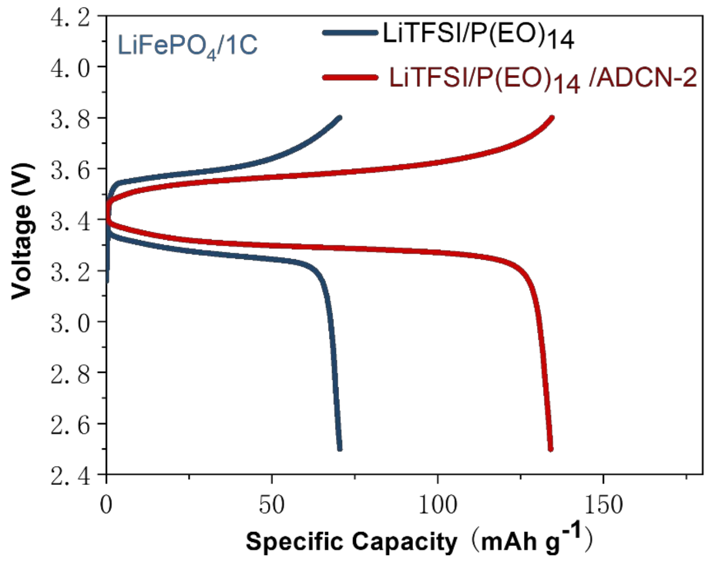

Fig. S5 Comparison of the charge-discharge curves of the LFP/LiTFSI/$(PEO)_{14}$/Li and LFP/LiTFSI/$(PEO)_{14}$/ADCN-2/Li at 1C

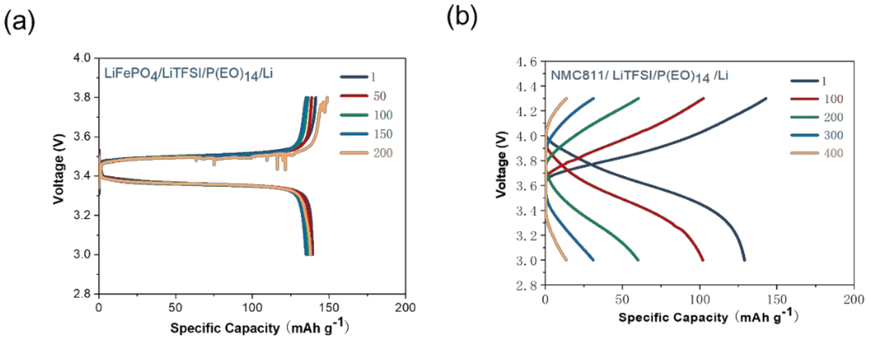

Fig. S6 Charge-discharge curves of the (**a**) LFP/LiTFSI/$(PEO)_{14}$/Li and (**b**) NMC811 /LiTFSI/$(PEO)_{14}$/Li at various cycles

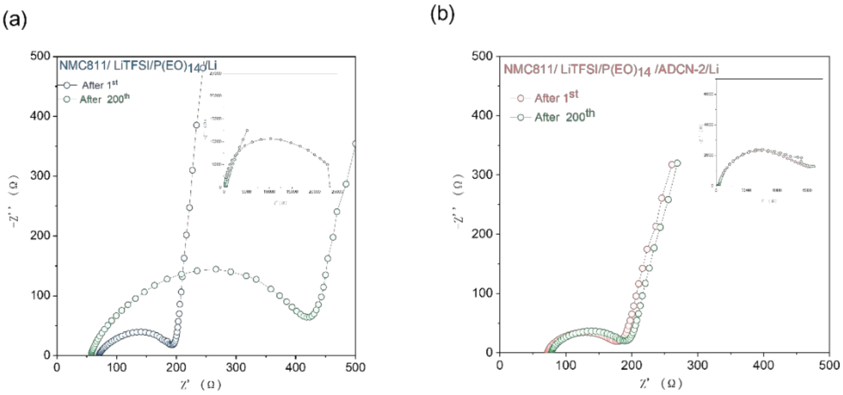

Fig. S7 EIS spectra of the NCM811/SPE/Li cells before and after 200 cycles for the (**a**) NMC811/ LiTFSI/$P(EO)_{14}$ and (**b**) NCM811/LiTFSI/$P(EO)_{14}$/ADCN-2/Li cells

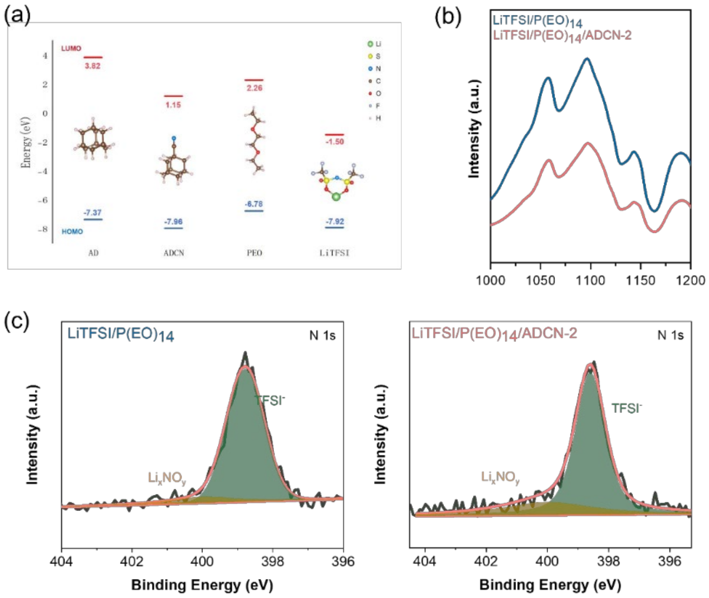

Fig. S8 (**a**) Calculated HOMO-LUMO energy of various compositions (**b**) XPS N1s spectra of the cycled cathodes (**c**) FTIR spectra of the cycled cathodes

Table S1 Crystallinity of polymer electrolytes

Polymer electrolytes	5_{g} (℃)	5_{m} (℃)	Δ) $_{m}$/(J/g)	χ_{c}/%
LiTFSI/$P(EO)_{14}$	-44.9	44.7	31.45	16.04
LiTFSI/$P(EO)_{14}$/ADCN-1	-45.9	44.6	26.26	13.40
LiTFSI/$P(EO)_{14}$/ADCN-2	-46.5	41.7	16.9	8.62
LiTFSI/$P(EO)_{14}$/ADCN-3	-45.0	43.8	18.86	9.62

Table S2 Comparison of the all-solid-state NMC/Li batteries with PEO-based electrolytes

Electrolyte components	Voltage-cutoff (V) And Cathode materials	Mass loading (mg cm^{-2})	Temp. (°C)	Cyclability	Initial specific capacity	Refs.
LiTFSI/$P(EO)_{10}$-15wt%Al_2O_3-0.5wt% $Mg(ClO_4)_2$	4.3 NMC811	3	55	0.1C 80 cycles ~73 %	0.1C 138.6 mAh g^{-1}	[S4]
LiTFSI/$P(EO)_{15}$-1wt%LiS_2	4.2 NMC811	2.1-2.5	50	0.2C 150 cycles 91.2%	0.2C 159.6 mAh g^{-1}	[S5]
LiTFSI/$P(EO)_{10}$-20wt%$Li_{3/8}Sr_{7/16}Ta_{3/4}Zr_{1/4}O_3$	4.3 NMC811	0.6	45	0.05C 120 cycles ~81.5%	0.05C 146 mAh g^{-1}	[S6]
LiTFSI/$P(EO)_{10}$-15wt%ZIF-8	4.3 NMC811	3	60	0.2C 50 cycles ~87.6%	0.2C 161.2 mAh g^{-1}	[S7]
LiTFSI/$P(EO)_{16}$- $La_2Zr_2O_7$	4.2 NCM811	/	45	0.1C 50 cycles, ~75.7%	0.1C 177mAh g^{-1}	[S8]
LiTFSI/$P(EO)_{10}$-45wt%PEGdMA	4.3 NCM622	/	40	1C 50 cycles ~90%	1C 145 mAh g^{-1}	[S9]
LiTFSI/$P(EO)_{16}$-CuF_2	4.1 $LiNi_{0.83}Co_{0.12}Mn_{0.05}O_2$ (NCM83)	2	30	0.6C 500 cycles, ~71%	0.6C 147.7 mAh g^{-1}	[S10]
LiTFSI/$P(EO)_{10}$-1wt%C6H5-CF3	4.3 NMC811	2	60	0.2 C 160 cycles ～88%	0.2C 141.2 mAh g-1	[S11]
hc-$Li_{2+x}Zr_{1-x}In_xCl_6$ ($0.3 \leq x \leq 1$)	2.82−4.42 NMC811	/	25	1 C 500 cycles ~74%	1 C 169 mAh g^{-1}	[S12]
$Li_3(CB_{11}H_{12})_2(CB_9H_{10})$	4 NMC811	/	60	0.5 C 350 cycles ～75%	0.5C 175 mAh g^{-1}	[S13]
$Li_{10}GeP_2S_{12}$	4.1 PS-LPO-NMC811	/	25	0.3C 250 cycles ~ 80%	0.3C 161 mAh g^{-1}	[S14]
LiTFSI/$P(EO)_{14}$-5wt%$C_{11}H_{15}N$	4.3 NMC811	1-2	45	0.3 C 1000 cycles ～80%	0.3C 143.4 mAh g^{-1}	This work

Supplementary References

[S1] J. VandeVondele, M. Krack, F. Mohamed, M. Parrinello, T. Chassaing, et al. Quickstep: Fast and accurate density functional calculations using a mixed gaussian and plane waves approach. Comput. Phys. Commun. **167**(2), 103-128 (2005). https://doi.org/10.1016/j.cpc.2004.12.014

[S2] C. Lee, W. Yang, R. G. Parr. Development of the colle-salvetti correlation-energy formula into a functional of the electron density. Phys. Rev. B **37**(2), 785-789 (1988). https://doi.org/10.1103/PhysRevB.37.785

[S3] S. Goedecker, M. Teter, J. Hutter. Separable dual-space gaussian pseudopotentials. Phys. Rev. B **54**(3), 1703 (1996).

[S4] B. Xu, X. Li, C. Yang, Y. Li, N. S. Grundish, et al. Interfacial chemistry enables stable cycling of all-solid-state li metal batteries at high current densities. J. Am. Chem. Soc. **143**(17), 6542-6550 (2021). https://doi.org/10.1021/jacs.1c00752

[S5] O. Sheng, J. Zheng, Z. Ju, C. Jin, Y. Wang, et al. In situ construction of a lif-enriched interface for stable all-solid-state batteries and its origin revealed by cryo-tem. Adv. Mater. **32**(34), 2000223 (2020). https://doi.org/0.1002/adma.202000223

[S6] H. Xu, P.-H. Chien, J. Shi, Y. Li, N. Wu, et al. High-performance all-solid-state batteries enabled by salt bonding to perovskite in poly(ethylene oxide). Proc. Natl. Acad. Sci. **116**(38), 18815-18821 (2019). https://doi.org/10.1073/pnas.1907507116

[S7] J. Shen, Z. Lei, C. Wang. An ion conducting zif-8 coating protected peo based polymer electrolyte for high voltage lithium metal batteries. Chem. Engin. J. **447**(13), 7503 (2022). https://doi.org/https://doi.org/10.1016/j.cej.2022.137503

[S8] J. Ma, G. Zhong, P. Shi, Y. Wei, K. Li, et al. Constructing a highly efficient "solid–polymer–solid" elastic ion transport network in cathodes activates the room temperature performance of all-solid-state lithium batteries. Energy Environ. Sci. **15**(4), 1503-1511 (2022). https://doi.org/10.1039/d1ee03345j

[S9] G. Homann, L. Stolz, K. Neuhaus, M. Winter, J. Kasnatscheew. Effective optimization of high voltage solid-state lithium batteries by using poly(ethylene oxide)-based polymer electrolyte with semi-interpenetrating network. Adv. Funct. Mater. **30**(46), 2006289 (2020). https://doi.org/10.1002/adfm.202006289

[S10] Y. Wei, T. H. Liu, W. Zhou, H. Cheng, X. Liu, et al. Enabling all-solid-state li metal batteries operated at 30 °C by molecular regulation of polymer electrolyte. Adv. Energy Mater. **13**(10), (2023). https://doi.org/10.1002/aenm.202203547

[S11] C. Li, S. Zhou, L. Dai, X. Zhou, B. Zhang, et al. Porous polyamine/peo composite solid electrolyte for high performance solid-state lithium metal batteries. J. Mater. Chem. A **9**(43), 24661-24669 (2021). https://doi.org/10.1039/d1ta04599g

[S12] K. Wang, Z. Gu, H. Liu, L. Hu, Y. Wu, et al. High-humidity-tolerant chloride

solid-state electrolyte for all-solid-state lithium batteries. Adv. Sci. (2024). https://doi.org/10.1002/advs.202305394

[S13] H. Braun, R. Asakura, A. Remhof, C. Battaglia. Hydroborate solid-state lithium battery with high-voltage nmc811 cathode. ACS Energy Lett. **9**(2), 707-714 (2024). https://doi.org/10.1021/acsenergylett.3c02117

[S14] J. Liang, Y. Zhu, X. Li, J. Luo, S. Deng, et al. A gradient oxy-thiophosphate-coated ni-rich layered oxide cathode for stable all-solid-state li-ion batteries. Nat. Commun. **14**(1), (2023). https://doi.org/10.1038/s41467-022-35667-7
